# Hydro­static behaviour of highly inert Fomblin and Halocarbon fluids as pressure-transmitting media in high-pressure experiments

**DOI:** 10.1107/S1600576725000342

**Published:** 2025-02-01

**Authors:** Klemen Motaln, Erik Uran, Nico Giordano, Simon Parsons, Matic Lozinšek

**Affiliations:** a Jožef Stefan Institute Jamova cesta 39 1000Ljubljana Slovenia; b Jožef Stefan International Postgraduate School Jamova cesta 39 1000Ljubljana Slovenia; c Deutsches Elektronen-Synchrotron DESY Notkestr. 85 22607Hamburg Germany; d EaStCHEM School of Chemistry and Centre for Science at Extreme Conditions University of Edinburgh King’s Buildings, W. Mains Road EdinburghEH9 3FJ United Kingdom; The University of Western Australia, Australia

**Keywords:** pressure-transmitting media, ruby fluorescence measurements, high-pressure crystallography, single-crystal X-ray diffraction, diamond anvil cells, DACs, hydro­staticity

## Abstract

A pressure-transmitting medium (PTM) plays an important role in diamond anvil cell (DAC) experiments as it ensures that the sample is exposed to hydro­static pressure. Although PTMs that are liquids under ambient conditions are the easiest to handle and load, the selection of chemically inert liquid media with established hydro­static properties is limited. To widen the choice of highly inert PTMs for high-pressure experiments, the hydro­static behaviours of Fomblin Z60, Fomblin Z25, Fomblin Y LVAC 06/6 and Halocarbon Oil 11-14 were investigated. The ruby fluorescence method was used to monitor the evolution of pressure gradients across the DAC sample chamber during compression and decompression. Fomblin Z60 and Fomblin Z25 perfluoro­polyethers, which are hydro­static to 1.7 and 1.5 GPa, respectively, exhibited the best hydro­static performance, followed by Halocarbon Oil 11-14 with a limit of 1.2 GPa, whereas the non-hydro­static behaviour of Fomblin Y LVAC 06/6 was observed above only 0.6 GPa.

## Abstract 2

Hydro­static properties of highly inert perhalogenated fluids were investigated using ruby fluorescence measurements.

## 1. Introduction

The application of high-pressure research is increasingly prevalent across a diverse range of scientific disciplines, including physics, chemistry, materials research and geoscience (Hemley, 2000 ▸; Duffy, 2005 ▸; Zhang *et al.*, 2017 ▸; Mao *et al.*, 2018 ▸; Miao *et al.*, 2020 ▸; Moggach & Oswald, 2020 ▸). In the field of chemistry, advances in laboratory-based diffractometer technology, access to dedicated high-pressure synchrotron beamlines and the increased availability of diamond anvil cells (DACs) have prompted a surge in investigations focusing on the analysis of pressure-induced structural changes by single-crystal X-ray diffraction (SCXRD) (Katrusiak, 2008 ▸, 2019 ▸; Tidey *et al.*, 2014 ▸; Zakharov & Boldyreva, 2019 ▸). In such investigations, it is highly desirable that uniform pressure is exerted on all of the surfaces of the crystal (Miletich *et al.*, 2000 ▸). The presence of non-hydro­static conditions can influence the occurrence of pressure-induced phase transitions and may also induce structural changes that are not necessarily related solely to pressure, but rather also to shear strain and deviatoric and uniaxial stresses (Takemura, 2021 ▸). It is also well established that non-hydro­static conditions can be responsible for degradation and amorphization of single crystals (Gillet *et al.*, 1995 ▸; Machon *et al.*, 2003 ▸). Furthermore, precise information regarding the pressure in the DAC experiments is vital not only for ensuring experimental accuracy and reproducibility but also for facilitating comparisons with the results of theoretical modelling that assume hydro­static compression (Takemura, 2021 ▸).

Hydro­static conditions within the sample chamber of the DAC are achieved through the use of a pressure-transmitting medium (PTM). A variety of PTMs are currently utilized for high-pressure experiments, encompassing materials that are either gaseous, liquid or solid under ambient conditions. Liquid or solidified gases typically demonstrate the highest hydro­static limits. Among these, helium, neon and nitro­gen are popular for accessing pressures beyond 10 GPa and for their high degree of inertness (Klotz *et al.*, 2009 ▸). Nevertheless, the loading of gases typically requires the use of specialized gas-loading systems, which are mainly accessible at synchrotron facilities or specially equipped laboratories (Rivers *et al.*, 2008 ▸; Kurnosov *et al.*, 2008 ▸). The commonly utilized PTMs employed in high-pressure SCXRD experiments are organic solvent mixtures, such as 5:1 iso­pentane–*n*-pentane and 4:1 methanol–ethanol, which are usually straightforward to handle and load and can remain hydro­static up to 7.4 and 10.5 GPa, respectively (Klotz *et al.*, 2009 ▸). A liquid PTM is not suitable if it dissolves or reacts with the sample. In the case of porous materials, the PTM may also penetrate the pores, changing the chemical composition and structure of the material (McKellar & Moggach, 2015 ▸; Collings & Goodwin, 2019 ▸). Reactions with the sample can be avoided using a chemically inert PTM; popular examples include silicone oils, Daphne series oils (7373, 7474 and 7575), and Fluorinert fluids and their mixtures. Among these, Daphne 7474 has been found to exhibit one of the highest hydro­static limits of approximately 4 GPa (Murata *et al.*, 2008 ▸; Klotz *et al.*, 2009 ▸), whereas the hydro­static limits of other Daphne oils studied are lower (Varga *et al.*, 2003 ▸; Sidorov & Sadykov, 2005 ▸; Klotz *et al.*, 2009 ▸; Staško *et al.*, 2020 ▸). Among Fluorinerts, which are perfluorinated compounds and thus offer the greatest chemical inertness, the highest hydro­static limit of 2.3 GPa was reported for the Fluorinert FC84–FC87 1:1 mixture (Sidorov & Sadykov, 2005 ▸; Klotz *et al.*, 2009 ▸), whereas the hydro­static limits of other examined Fluorinert PTMs typically lie below 2.2 GPa (Varga *et al.*, 2003 ▸; Sidorov & Sadykov, 2005 ▸; Torikachvili *et al.*, 2015 ▸). Another class of highly chemically inert fluids are the Fomblin series of perfluoro­polyether (PFPE) synthetic polymer liquid lubricants. Prior investigations into the hydro­static behaviour of one type of Fomblin oil, Fomblin Y HVAC 140/13, revealed that it is essentially non-hydro­static above 1 GPa (Koyama-Nakazawa *et al.*, 2007 ▸; Osakabe & Kakurai, 2008 ▸). Moreover, perhalogenated PTMs that contain no hydrogen can be employed for high-pressure neutron experiments, as these media typically exhibit only very small neutron incoherent scattering (Varga *et al.*, 2003 ▸; Sidorov & Sadykov, 2005 ▸).

To widen the selection of highly inert fluids which could serve as PTMs and demonstrate satisfactory hydro­static performance, Fomblin Z60, Fomblin Z25 and Fomblin Y LVAC 06/6 perfluoro­polyethers, as well as the poly(chloro­tri­fluoro­ethyl­ene) Halocarbon Oil 11-14, have been investigated in this work. The hydro­static behaviour of selected fluids was studied by the ruby fluorescence technique, which enables the determination of the pressure distribution experienced by ruby balls located at different positions within a DAC (Piermarini *et al.*, 1973 ▸; Klotz *et al.*, 2009 ▸). The specific fluids were selected because of their exceptional inertness, which is reflected in the fact that Fomblin Z25 can be used for mounting single crystals of highly reactive and strongly oxidizing noble-gas compounds (Lozinšek *et al.*, 2021 ▸; Motaln *et al.*, 2024 ▸).

## 2. Experimental

**Figure 1:**
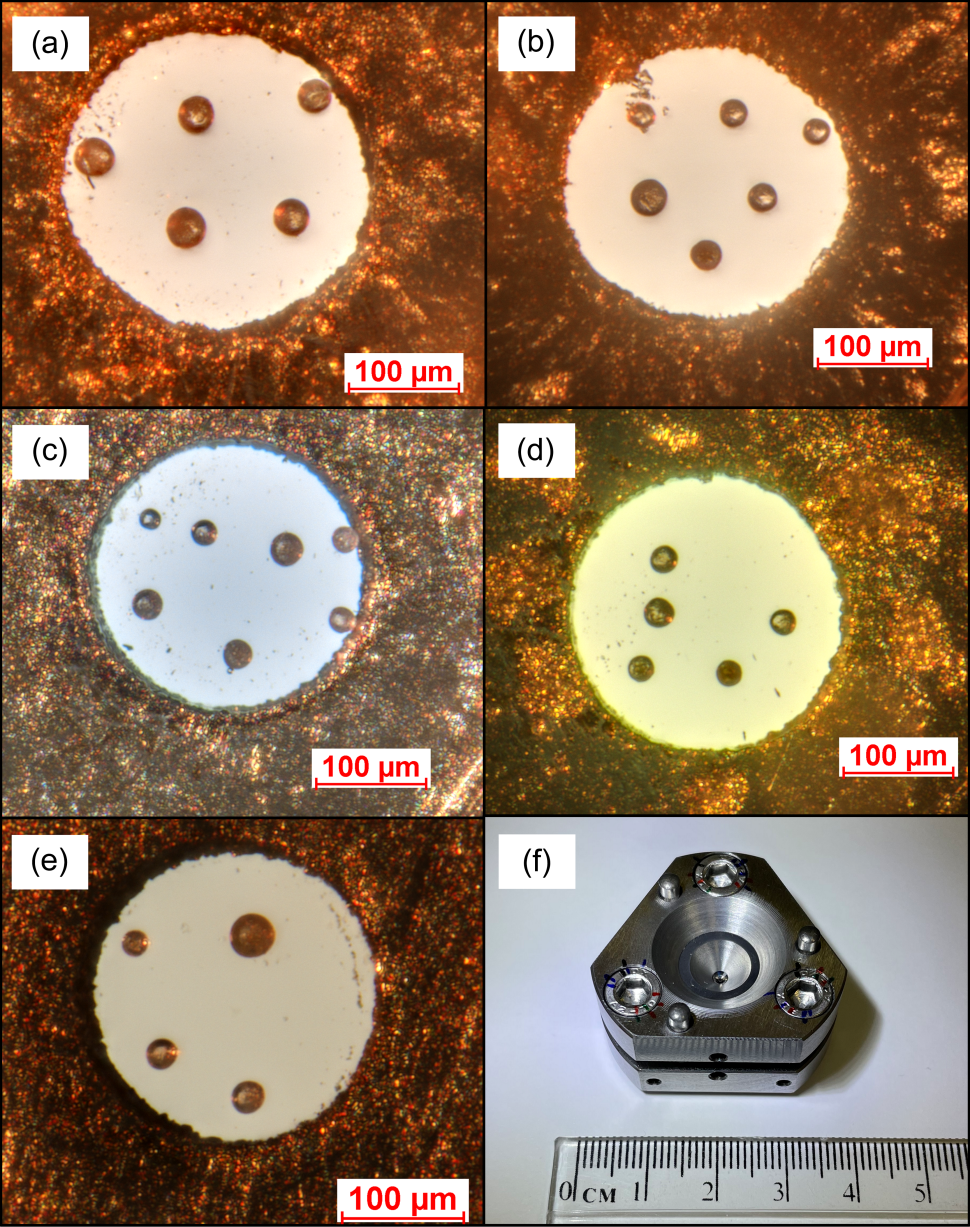
DAC pressure chambers showing the distribution of the ruby balls loaded with the following fluids: (*a*) Fomblin Z60, (*b*) Fomblin Z25, (*c*) Fomblin Y LVAC 06/6, (*d*) Halocarbon Oil 11-14 and (*e*) N_2_. The differences in colour are an artefact of illumination. (*f*) Merrill–Bassett DAC, as used in this study.

Perfluoro­polyethers Fomblin Z60 (Synquest), Fomblin Z25 (Synquest), Fomblin Y LVAC 06/6 (Aldrich) and the poly(chloro­tri­fluoro­ethyl­ene) Halocarbon Oil 11-14 (Halocarbon Products Corp.) were used as supplied (Fig. S1 of the supporting information). The Raman (Fig. S2) and attenuated total reflectance IR spectra (Fig. S3) of the fluids, along with the relevant measurement details, are provided in the supporting information. The inertness of the fluids was tested by bringing them into contact with XeF_2_ in an inert atmosphere: no reaction or evolution of Xe gas was observed in any case. In all experiments, Merrill–Bassett type cell bodies (Fig. 1 ▸) (Merrill & Bassett, 1974 ▸) were paired with Boehler–Almax design type Ia or IIas diamonds (Boehler & Hantsetters, 2004 ▸) with 600 µm diameter culets and tungsten carbide seats (Moggach *et al.*, 2008 ▸) (Almax easyLab). Inconel X750 (Goodfellow, Ni74/Cr15/Fe7/Ti/Al/Nb, annealed) plates, with a starting thickness of 250 µm, were employed as gaskets and pre-indented to a thickness of approximately 100 µm. Subsequently, the gasket holes with a 250 µm diameter were drilled using a spark eroder (LOTO-eng SEC-400). Prior to use, the gaskets were cleaned in an ultrasonic bath containing ethanol. In order to load the cells, 4–7 ruby balls [BETSA, Al_2_O_3_:Cr^3+^, 3600 p.p.m. Cr^3+^, 3–50 µm mean diameter (Chervin *et al.*, 2001 ▸)] were placed on the culet of the upper diamond, with the investigated PTM placed within the gasket hole. The DAC was then closed, ensuring that no air bubbles remained trapped within. Care was taken to ensure that the ruby balls were distributed throughout the sample chamber (Fig. 1 ▸).

Ruby fluorescence spectra (Syassen, 2008 ▸) were recorded at room temperature (23 ± 1 °C) using a Bruker Sentera II confocal Raman microscope. Rubies were excited by the 532 nm laser with a power output of 0.5 mW. The fluorescence spectra were recorded in the range 635–725 nm by accumulation of ten acquisitions with an exposure time of 1 ms per acquisition. The spectrum of each ruby in the DAC was measured four times in the course of each measurement cycle. The first measurement cycle of the spectra was conducted 15 min after the pressure in the cell was increased, with an additional measurement cycle following at least 24 h later in order to account for mechanical equilibration and creep effects (Piermarini *et al.*, 1973 ▸). If a significant change in the measured pressure was observed, the measurement cycle was repeated one day later until no further change was observed. Further compression was only performed once the pressure at various points within the cell fully stabilized. The fluorescence spectra measured just before a new round of compression were used for the calculation of pressure deviation. When differences of >1 GPa were detected between different ruby balls, typically at pressures of 7–9 GPa, the cells were gradually decompressed in a manner analogous to the compression cycle, with measurements taken at each step of the decompression.

Prior to spectrum analysis, background subtraction was performed in the Bruker *Opus* 8.7 software suite, utilizing a concave rubber band correction with three iterations and 1024 baseline points. The spectra were fitted with Pearson VII functions in the *Fityk* program (Wojdyr, 2010 ▸), with optimization by the *VAR2* method (Vlček & Lukšan, 2006 ▸) from the *NLopt* library (Johnson, 2007 ▸). Under markedly non-hydro­static conditions, a shoulder began to emerge in the *R*_1_ line which significantly compromised the fit. This issue was addressed by incorporating a third Pearson VII function into the optimization, which enabled the fitting of this shoulder and led to an improvement of the overall fit. The fitting yielded the position of the *R*_1_ and *R*_2_ peaks and their respective full width at half-maximum values (fwhm). The separation between *R*_1_ and *R*_2_ peak positions was calculated as Δ*R* = *R*_1_ − *R*_2_. The reference position of the *R*_1_ line under ambient conditions was obtained by measuring the fluorescence spectra of five rubies kept under ambient conditions and adhered to a microscope slide by the use of Fomblin Z25 twice prior to and twice after each measurement cycle. The spectra were processed and analysed in accordance with the aforementioned methodology, and the mean value of all extracted *R*_1_ peak positions was employed as the reference wavelength of the *R*_1_ line in subsequent pressure calculations during the corresponding measurement cycle. Pressures were calculated with the Ruby2020 pressure gauge equation (Shen *et al.*, 2020 ▸).

The onset of non-hydro­static conditions was monitored by plotting the standard deviation of the measured pressures (σ*_P_*), the averaged values of Δ*R* and the fwhm of the *R*_1_ peaks as a function of the average pressure in the cell *P*_avg_, as previously outlined (Klotz *et al.*, 2009 ▸). The value of *P*_avg_ is the mean of all the pressures obtained in a single cycle of measurement, individually calculated from the position of the *R*_1_ peak of each ruby in the cell, each measured four times (see Section S3 in the supporting information). Similarly, the mean values of Δ*R* and fwhm of the *R*_1_ peak were obtained by averaging all the individual Δ*R* and fwhm(*R*_1_) values obtained in a single measurement cycle. The σ*_P_* value is calculated as the standard deviation of the mean pressures of all loaded rubies.

In order to benchmark the employed methodology, an experimental run utilizing N_2_ as the PTM, for which the hydro­static behaviour is well established (LeSar *et al.*, 1979 ▸; Klotz *et al.*, 2009 ▸), was also conducted. A Merrill–Bassett cell body with 600 µm culet type Ia Boehler–Almax diamond anvils was employed in conjunction with a stainless-steel gasket [with a thickness of 250 µm, preindented to approximately 70 µm with a 250 µm-diameter hole; Fig. 1 ▸(*e*)]. N_2_ was loaded into the DAC by submerging a partially opened cell that had been pre-loaded with ruby balls into a bath of liquid nitro­gen. Once the DAC had cooled to the temperature of liquid nitro­gen, it was closed and fully tightened, trapping the liquid nitro­gen within the sample chamber.

## 3. Results

**Figure 2:**
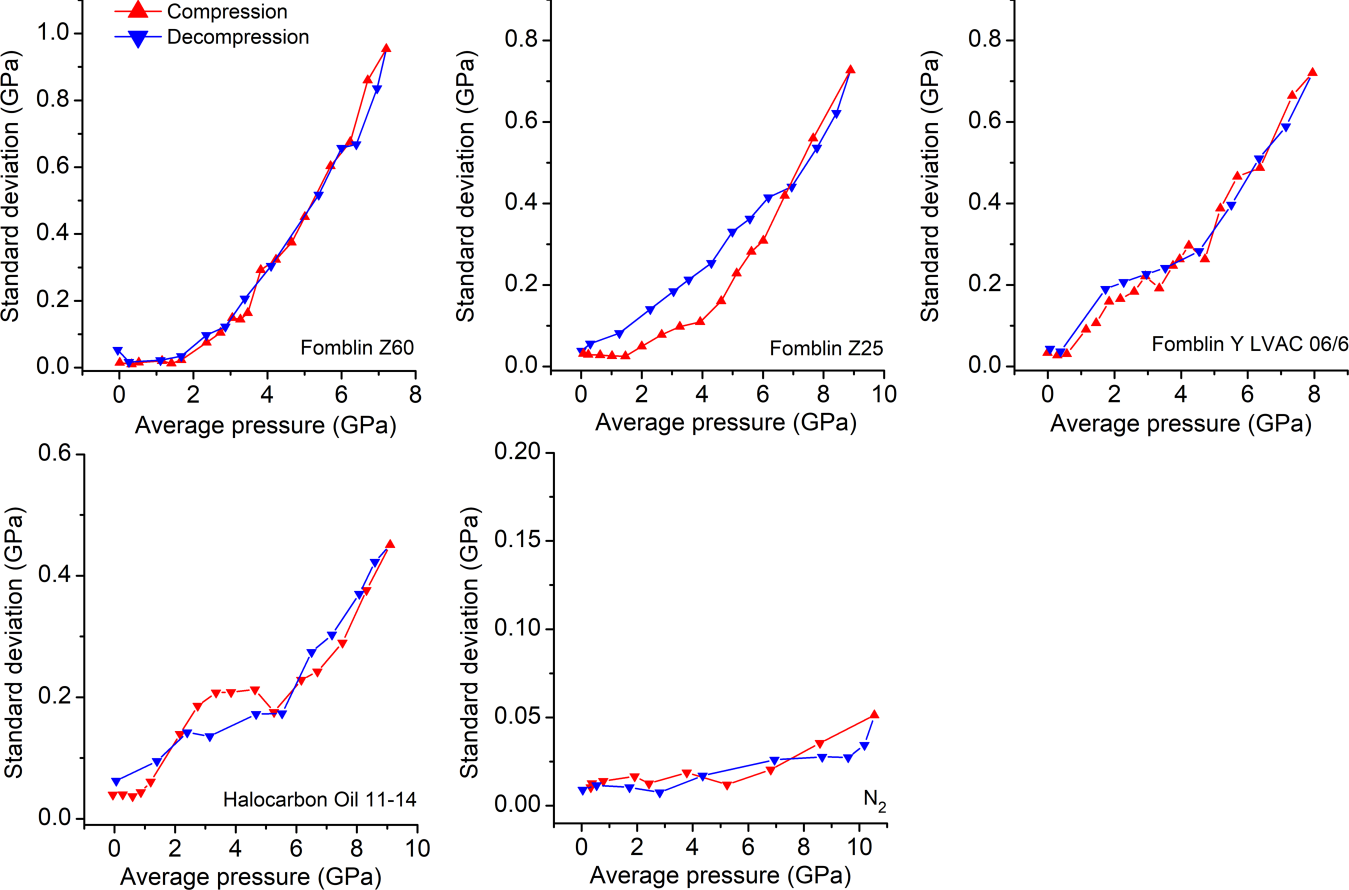
Plots displaying the pressure dependence of the standard deviation of pressure (σ*_P_*) from which the hydro­static limits of the fluids were deduced. As the pressure is increased, there is a sudden increase in σ*_P_*. This suggests that, at this point, the hydro­static limit is reached.

A reliable indicator for the appearance of non-hydro­staticity is the onset of an increase in the standard deviation of pressure (σ*_P_*) observed in the pressure dependence of σ*_P_* plots (Klotz *et al.*, 2009 ▸) (Figs. 2 ▸ and S4). The pressure dependences of Δ*R* and fwhm(*R*_1_) plots are depicted in the supporting information (Figs. S5 and S6).

Among the perhalogenated fluids examined, the highest hydro­static limit was observed for Fomblin Z60 and Z25 fluids, with the initial pressure gradients appearing above 1.7 and 1.5 GPa, respectively (Figs. 2 ▸ and S4; Table 1 ▸). These conclusions are corroborated by the pressure dependences of the Δ*R* and fwhm(*R*_1_) plots (Figs. S5 and S6), which also exhibit a large sudden increase in both parameters above the pressures identified in the pressure dependence of σ*_P_* plots (Fig. 2 ▸).

While σ*_P_* steadily rises as the pressure is increased in the non-hydro­static regime in the case of Fomblin Z60, more complex behaviour is seen for Fomblin Z25. Following the initial increase, the σ*_P_* value rises relatively slowly and nearly reaches a plateau at the interval between 1.5 and 4 GPa, followed by a more rapid increase at higher pressures. Such a pattern is not evident in the Δ*R* and fwhm(*R*_1_) plots, which show a pronounced increase in the values of both Δ*R* and fwhm(*R*_1_) as the pressure is increased above 1.5 GPa (Figs. S5 and S6).

Halocarbon Oil 11-14 exhibits a sharp rise in σ*_P_* above 1.2 GPa, suggesting that this is the hydro­static limit. However, as the pressure increase was continued in the non-hydro­static regime, the increase of σ*_P_* was considerably more gradual than in other media, reaching 0.45 GPa (5%) at the final pressure point of 9.1 GPa. Such a standard deviation was observed in other fluids at considerably lower pressures, specifically at 5.0 GPa for Fomblin Z60, above 6.7 GPa for Fomblin Z25 and at 5.7 GPa for Fomblin Y LVAC 06/6.

The poorest hydro­static performance was demonstrated by Fomblin Y LVAC 06/6, where pressure gradients appeared above only 0.6 GPa.

A benchmark experiment with N_2_ as the pressure medium revealed a hydro­static behaviour up to a maximum attained pressure of 10.5 GPa, as shown from the pressure dependences of the σ*_P_*, Δ*R* and fwhm(*R*_1_) plots (Figs. 2 ▸, S4, S5 and S6). In addition, the pressure dependence plots of Δ*R* and fwhm(*R*_1_) show a slight decrease with *P*_avg_. These results are consistent with the previous findings (Klotz *et al.*, 2009 ▸).

Pressure measurements were also conducted during the stepwise decompression of the cells, and the results were compared with compression cycles. For Fomblin Z60 and Fomblin Y, the pressure dependences of σ*_P_* during the compression and decompression cycles corresponded very closely, but in the case of Fomblin Z25 a clear hysteresis was observed. Similarly, hysteresis was also noted in the majority of the pressure dependences of the Δ*R* and fwhm(*R*_1_) plots, with the exception of the pressure dependences of Δ*R* for Fomblin Z60 and Halocarbon Oil 11-14, which show reasonable agreement between the compression and decompression. Moreover, the values of Δ*R* and fwhm(*R*_1_) obtained after decompression to ambient pressure were higher than the values measured at the start of the compression for all fluids. The increase in the final fwhm(*R*_1_) values in comparison with the initial ones ranged from 0.04 nm in the case of Fomblin Z60 to 0.17 nm in the case of Halocarbon Oil 11-14 (Fig. S5), whereas the Δ*R* values after decompression increased between 0.01 nm in the case of Fomblin Z60 and 0.04 nm in the case of Fomblin Y LVAC 06/6 when compared with the initial measurements taken at the beginning of the compression cycle (Fig. S6). In the absence of any observable increase in the Δ*R* and fwhm(*R*_1_) parameter values obtained at ambient pressure after decompression when N_2_ was used as a PTM and subjected to an even higher pressure, it can be surmised that these effects may be due to the prolonged exposure of rubies to non-homogenous pressure (Adams *et al.*, 1976 ▸).

## 4. Conclusions

In this study, the hydro­static behaviour of Fomblin Z60, Fomblin Z25, Fomblin Y LVAC 06/6 and Halocarbon Oil 11-14 under high-pressure conditions was investigated by following the pressure distribution across the sample chamber employing the ruby fluorescence technique, thereby expanding the selection of highly inert fluids for which the hydro­static behaviour was examined. Among these fluids, the highest hydro­static limit of 1.7 GPa has been established for Fomblin Z60. An onset of non-hydro­static behaviour was observed at 1.5 GPa for Fomblin Z25, at 0.6 GPa for Fomblin Y LVAC 06/6 and at 1.2 GPa for Halocarbon Oil 11-14. Although the hydro­static limits of the media investigated in this study are relatively low, they are nonetheless considered useful for high-pressure experiments with samples whose reactivity precludes the use of more conventional PTMs. This is supported by other recent results from our laboratories, whereby the use of Fomblin Z25 and Halocarbon Oil 11-14 as PTMs provided useful SCXRD data at pressures above 5 GPa and Halocarbon Oil 11-14 was used as the PTM in neutron powder diffraction measurements up to 5 GPa (Clough *et al.*, 2025 ▸). Moreover, by mixing two or more of the fluids examined in this study a PTM mixture with an even higher hydro­static limit might be obtained, as evidenced in the case of other well established PTM mixtures (*e.g.* Fluorinert FC84–FC87, methanol–ethanol, iso­pentane–*n*-pentane).

## Supplementary Material

Supporting figures and calculations. DOI: 10.1107/S1600576725000342/oc5044sup1.pdf

## Figures and Tables

**Table 1:** Selected physical properties of the evaluated halogenated fluids and their observed hydro­static limits The values for Fomblin Z and Fomblin Y fluids were sourced from the respective product data sheet brochures: Fomblin PFPE Lubricants, Syensqo, R 06/2017, version 2.2, and Fomblin PFPE Lubes for Vacuum Applications, Syensqo, R 10/2017, version 2.7, respectively.

	Fomblin Z60	Fomblin Z25	Fomblin Y LVAC 06/6	Halocarbon Oil 11-14
Average molecular weight (g mol^−1^)	21500	17100	1800	–
Density at 20 °C (g cm^−3^)	1.85	1.85	1.88	1.90†
Kinematic viscosity at 20 °C (cSt)	600	223	64	–
Pour point (°C)	−63	−75	−50	–
Hydro­static limit (GPa)	≃1.7	≃1.5	≃0.6	≃1.2

† Density was determined at 20 °C using a pycnometer.

## Data Availability

The supporting information includes figures depicting the vibrational spectra of the evaluated fluids and descriptions of their acquisitions, the procedure used for the calculation of the parameter values at each measurement cycle, and plots showing the pressure dependences of the σ_*P*_, Δ*R* and fwhm(*R*_1_) values for the PTMs studied. A full cohort of raw experimental data and processed, fitted spectra is available upon request.
