# Supplementary material for: Hydro­static behaviour of highly inert Fomblin and Halocarbon fluids as pressure-transmitting media in high-pressure experiments
Source: J Appl Crystallogr. 2025 Feb 1;58(Pt 1):221–6. doi: 10.1107/S1600576725000342 (PMC11798517; doi:10.1107/S1600576725000342)
Supplement: Supplementary file 1 [file j-58-00221-sup1.pdf]

JOURNAL OF APPLIED CRYSTALLOGRAPHY

Volume 58 (2025)

Supporting information for article:

Hydrostatic behaviour of highly inert Fomblin and Halocarbon fluids as pressure-transmitting media in high-pressure experiments

Klemen Motaln, Erik Uran, Nico Giordano, Simon Parsons and Matic Lozinšek

S1. Experimental details – Materials

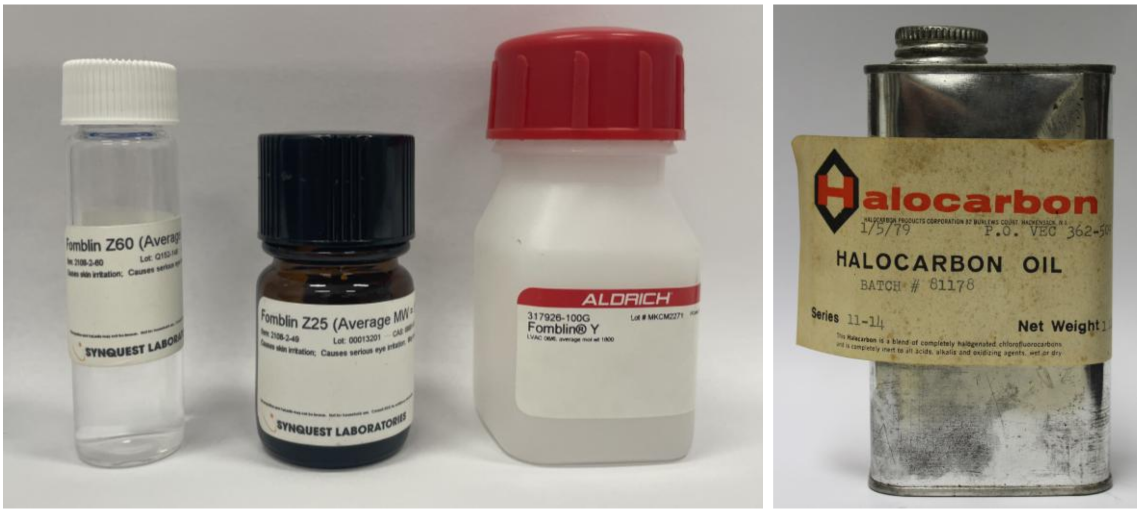

Figure S1 Perfluoropolyether fluids Fomblin Z60 (Synquest Laboratories; Item: 2108-2-60; Lot: Q152-148; CAS: 69991-61-3), Fomblin Z25 (Synquest Laboratories; Item: 2108-2-49; Lot: 00013201; CAS: 69991-61-3), Fomblin Y LVAC 06/6 (Aldrich; Item: 317926; Lot: MKCM2271; CAS: 69991-67-9) and the poly(chlorotrifluoroethylene) Halocarbon Oil 11-14 (Halocarbon Products Corp.; Batch: 81178) used in this work.

S2. Experimental details – Vibrational spectroscopy

S2.1. Raman spectroscopy

Ambient condition Raman spectra of the fluids (Figure S2) were recorded using a Bruker Senterra II confocal Raman microscope with a 785-nm emission line operating at a power output of 100 mW. Attempts to record the Raman spectra of fluids placed directly onto glass microscope slides resulted in the acquisition of spectra with a strong, broad signal of glass in the range 1000–2000 cm^{-1} and a maximum at ~1380 cm^{-1}. Therefore, a sheet of aluminium foil, which produces a minimal Raman background signal, was placed on the microscope slide, and the fluid drops were deposited on the foil using a dropper pipette. The spectra were recorded in the 50–3630 cm^{-1} range with a resolution of 4 cm^{-1} by accumulation of two acquisitions with an exposure time of 20 s per acquisition. The background was subtracted from the spectra using the Bruker *Opus* 8.7 software suite, employing three iterations of a concave rubber band correction with 64 baseline points.

S2.2. ATR-Infrared spectroscopy

Infrared spectra (Figure S3) were measured on a Bruker Alpha II FT-IR spectrometer, equipped with a Platinum Diamond-ATR sampling module, which was operated within an N_2 atmosphere glovebox (Vigor SG1500/750E). The spectra were recorded in the range of 400–4000 cm^{-1} with a resolution of 4 cm^{-1} and 24 scans were taken per measurement.

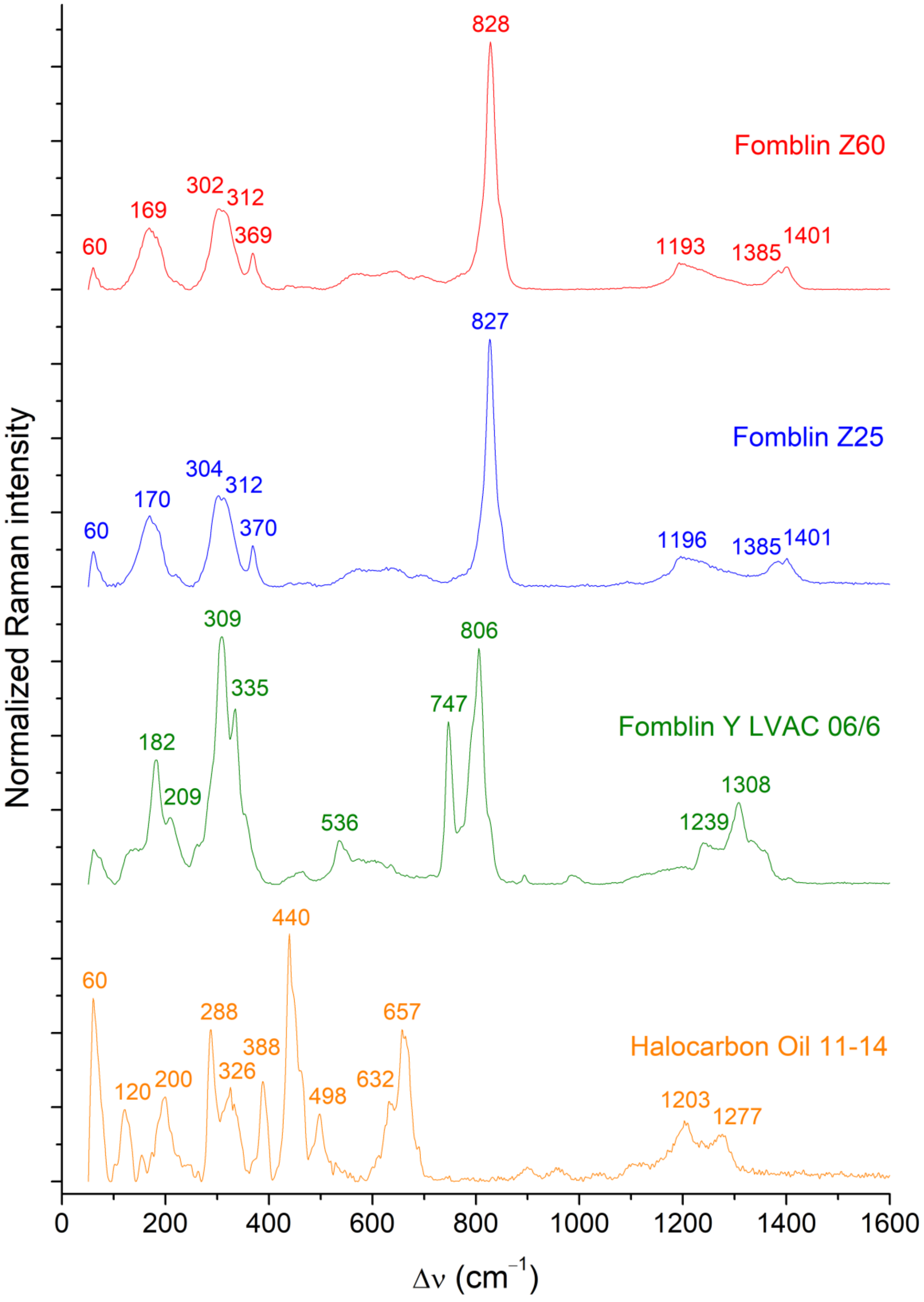

Figure S2 Ambient condition Raman spectra of the inert perhalogenated fluids evaluated in this work (Fomblin Z60, Fomblin Z25, Fomblin Y LVAC 06/6, and Halocarbon Oil 11-14). The spectra show no additional bands in the region 1600–3630 cm^{-1}.

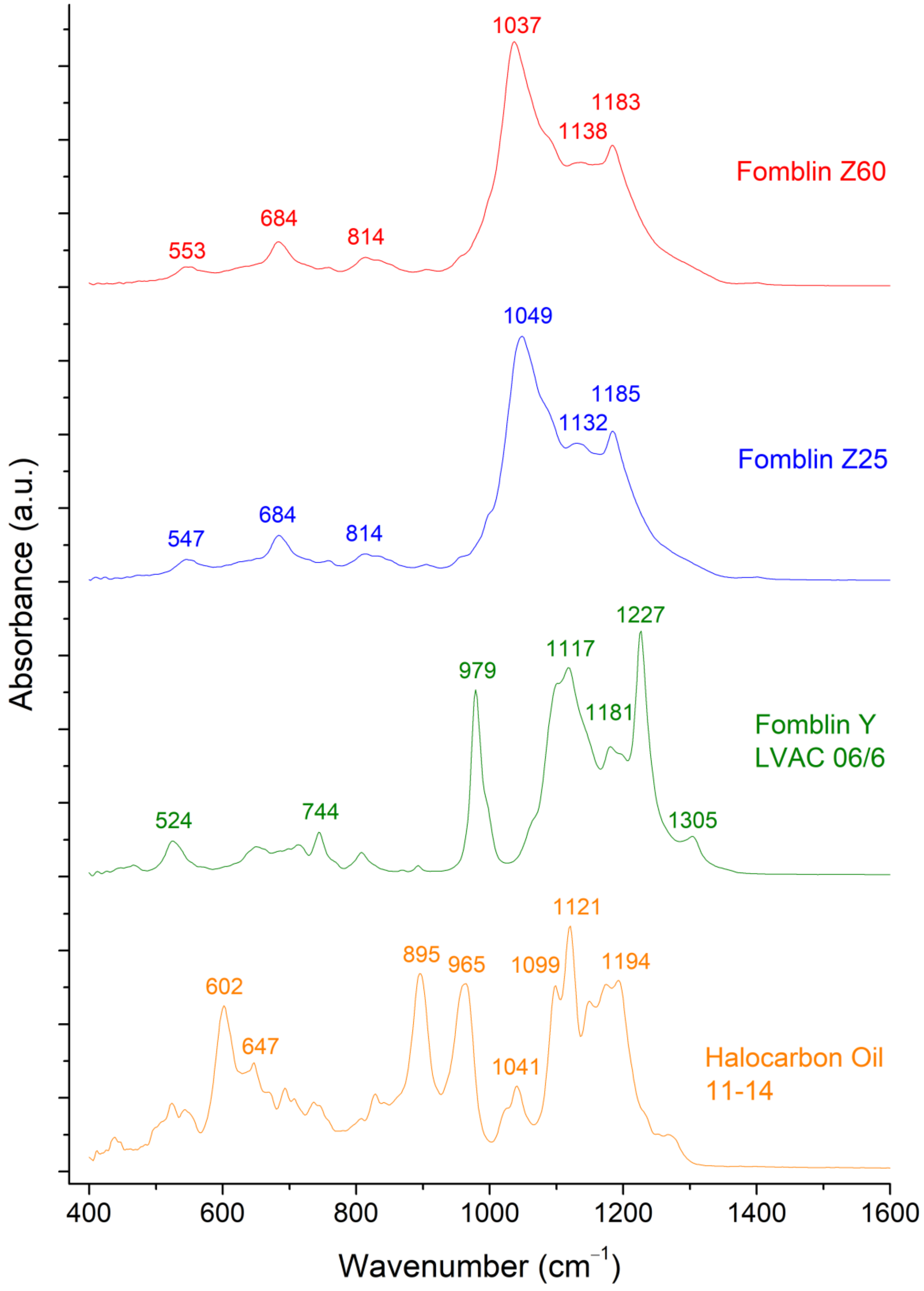

Figure S3 ATR-IR spectra of the inert perhalogenated fluids evaluated in this work (Fomblin Z60, Fomblin Z25, Fomblin Y LVAC 06/6, and Halocarbon Oil 11-14). The spectra show no additional features in the range 1600–4000 cm^{-1}.

S3. Experimental details – Calculations

The mean pressure within a cell at a specific measurement cycle was calculated as:

$$P_{\text{avg}} = \frac{1}{4n}\sum_{i=1}^{n}\sum_{j=1}^{4} P_{ij}$$

where n is the number of rubies and P_{ij} represents the pressure measured on the i-th ruby at the j-th acquisition, each individually calculated from the position of the respective $(R_1)_{ij}$ using the Ruby2020 pressure gauge equation. Number of rubies varied from 4 to 7 between the different experiments whereas each measurement cycle consisted of four separate spectra acquisitions at each ruby.

Standard deviation of the pressure in a specific measurement cycle was calculated as:

$$\sigma_P = \sqrt{\frac{\sum_{i=1}^{n}(\bar{P}_i - P_{\text{avg}})^2}{n}}$$

where n is the number of rubies and $\bar{P}_i$ represents the mean pressure at the i-th ruby calculated as the average of four acquisitions, for which the pressure was individually calculated from the position of the respective $(R_1)_{ij}$ using the Ruby2020 pressure gauge equation:

$$\bar{P}_i = \frac{1}{4}\sum_{j=1}^{4} P_{ij}$$

Mean full width at half maximum of the R_1 line, fwhm(R_1), and the mean difference between the positions of the R_1 and R_2 lines, $\Delta R = R_1 - R_2$, at a specific measurement cycle were calculated as:

$$\text{fwhm}(R_1) = \frac{1}{4n}\sum_{i=1}^{n}\sum_{j=1}^{4} \text{fwhm}(R_1)_{ij}$$

$$\Delta R = \frac{1}{4n}\sum_{i=1}^{n}\sum_{j=1}^{4} \Delta R_{ij}$$

where n is the number of rubies and $\text{fwhm}(R_1)_{ij}$ and ΔR_{ij} represent the fwhm of the R_1 line and ΔR values in the fluorescence spectrum of the i-th ruby at the j-th acquisition, respectively.

S4. Results

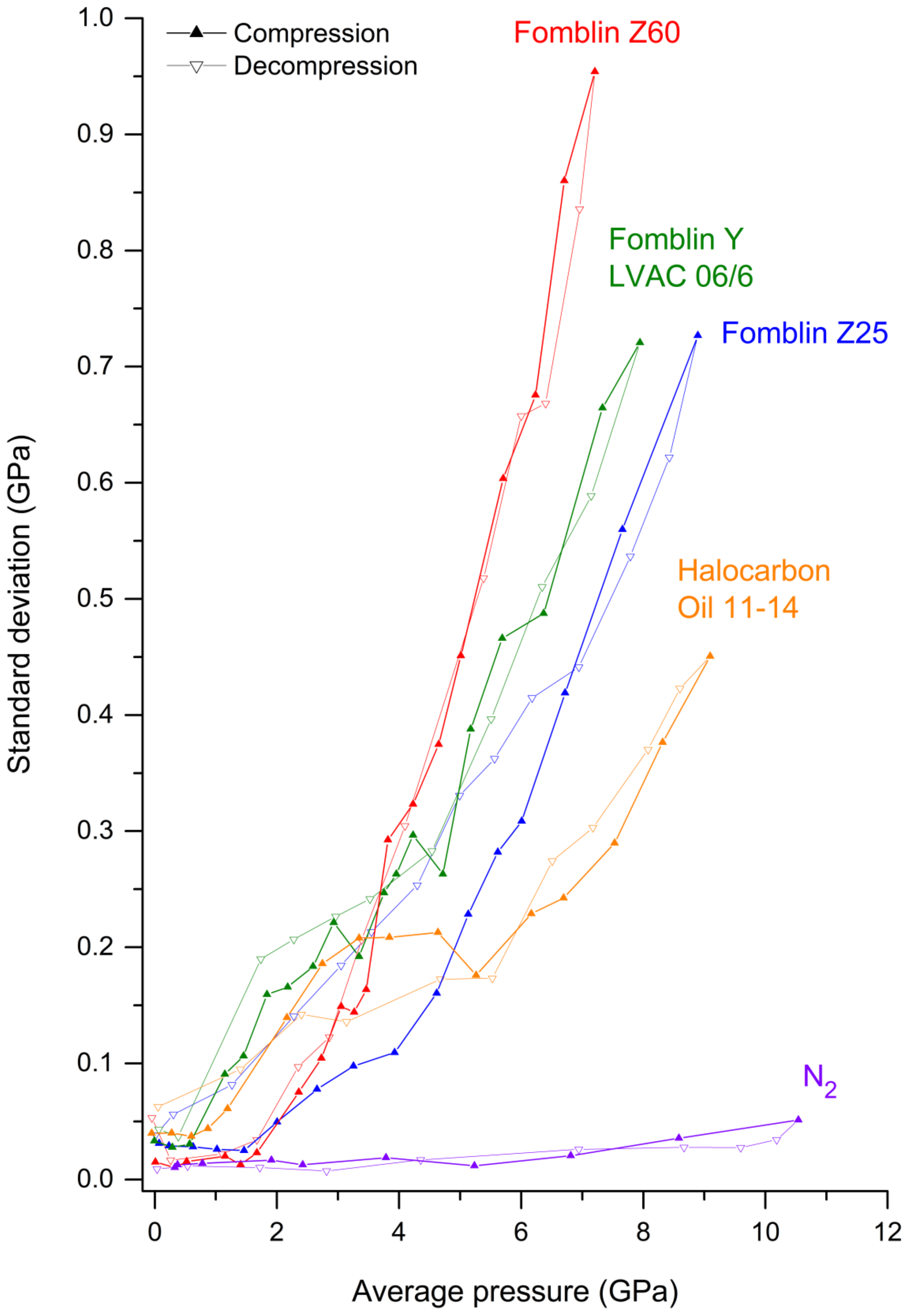

Figure S4 Pressure dependence of the standard deviation of pressure (σ_P) plot for Fomblin Z60, Fomblin Z25, Fomblin Y LVAC 06/6, Halocarbon Oil 11-14, and nitrogen.

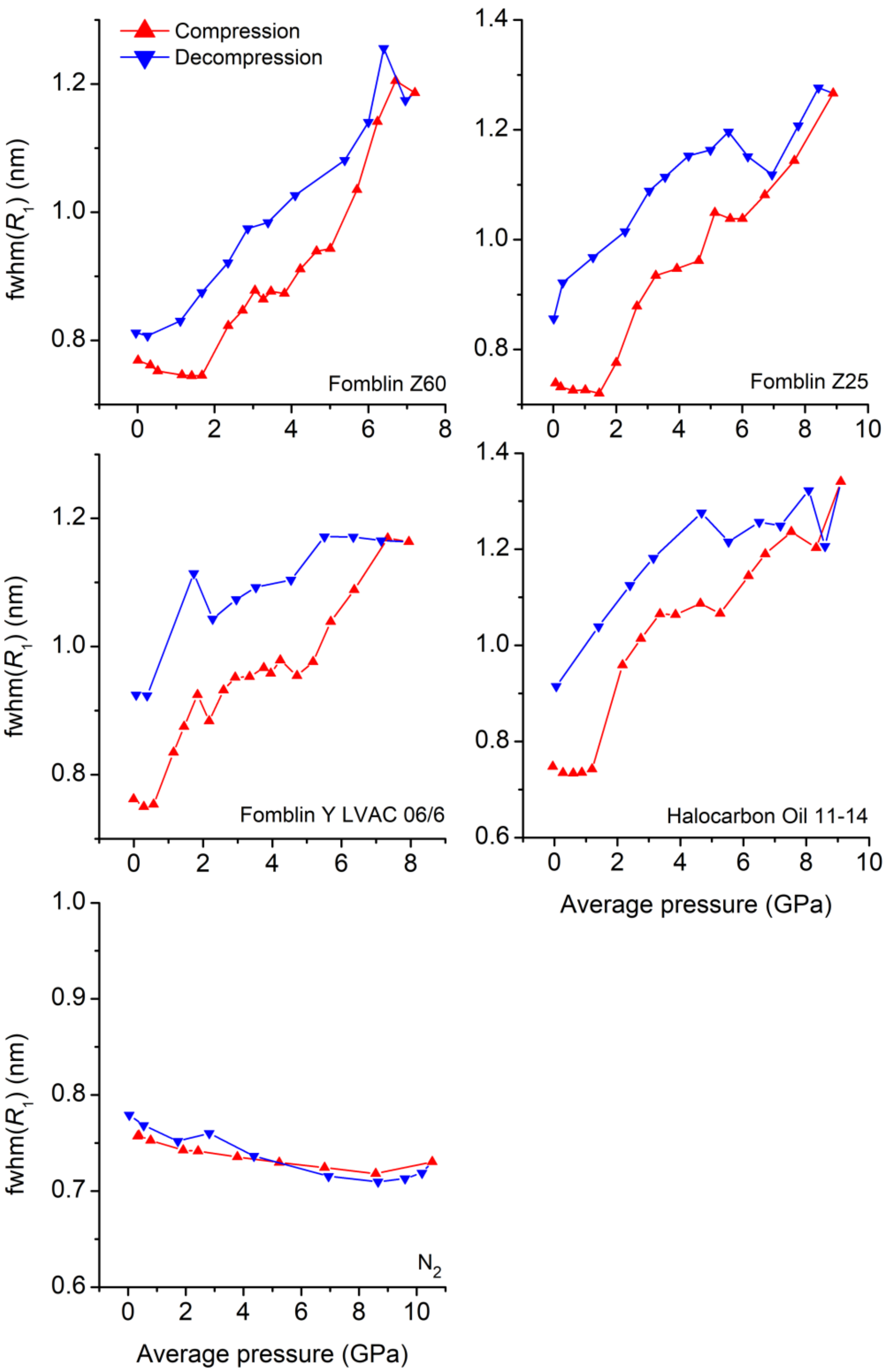

Figure S5 Pressure dependence of fwhm(R_1) plots for Fomblin Z60, Fomblin Z25, Fomblin Y LVAC 06/6, Halocarbon Oil 11-14, and nitrogen.

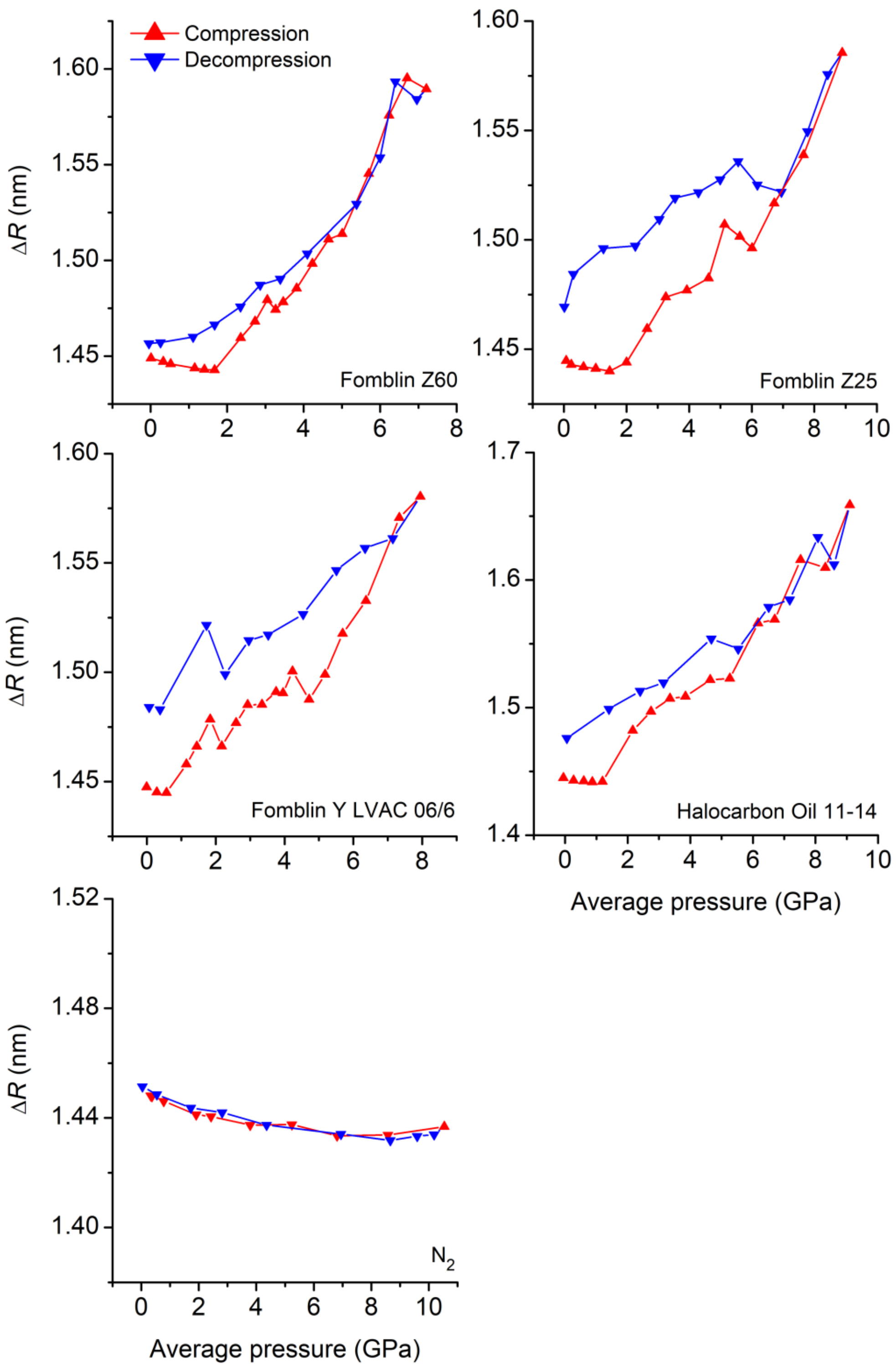

Figure S6 Pressure dependence of Δ*R* plots for Fomblin Z60, Fomblin Z25, Fomblin Y LVAC 06/6, Halocarbon Oil 11-14, and nitrogen.
